# Supplementary material for: Integrin-linked kinase controls retinal angiogenesis and is linked to Wnt signaling and exudative vitreoretinopathy
Source: Nat Commun. 2019 Nov 20;10:5243. doi: 10.1038/s41467-019-13220-3 (PMC6868140; doi:10.1038/s41467-019-13220-3)
Supplement: Supplementary file 1 — Supplementary Information [file 41467_2019_13220_MOESM1_ESM.pdf]

Supplementary Information

Integrin-linked kinase controls retinal angiogenesis and is linked to Wnt signaling and exudative vitreoretinopathy

Park, Yamamoto et al.

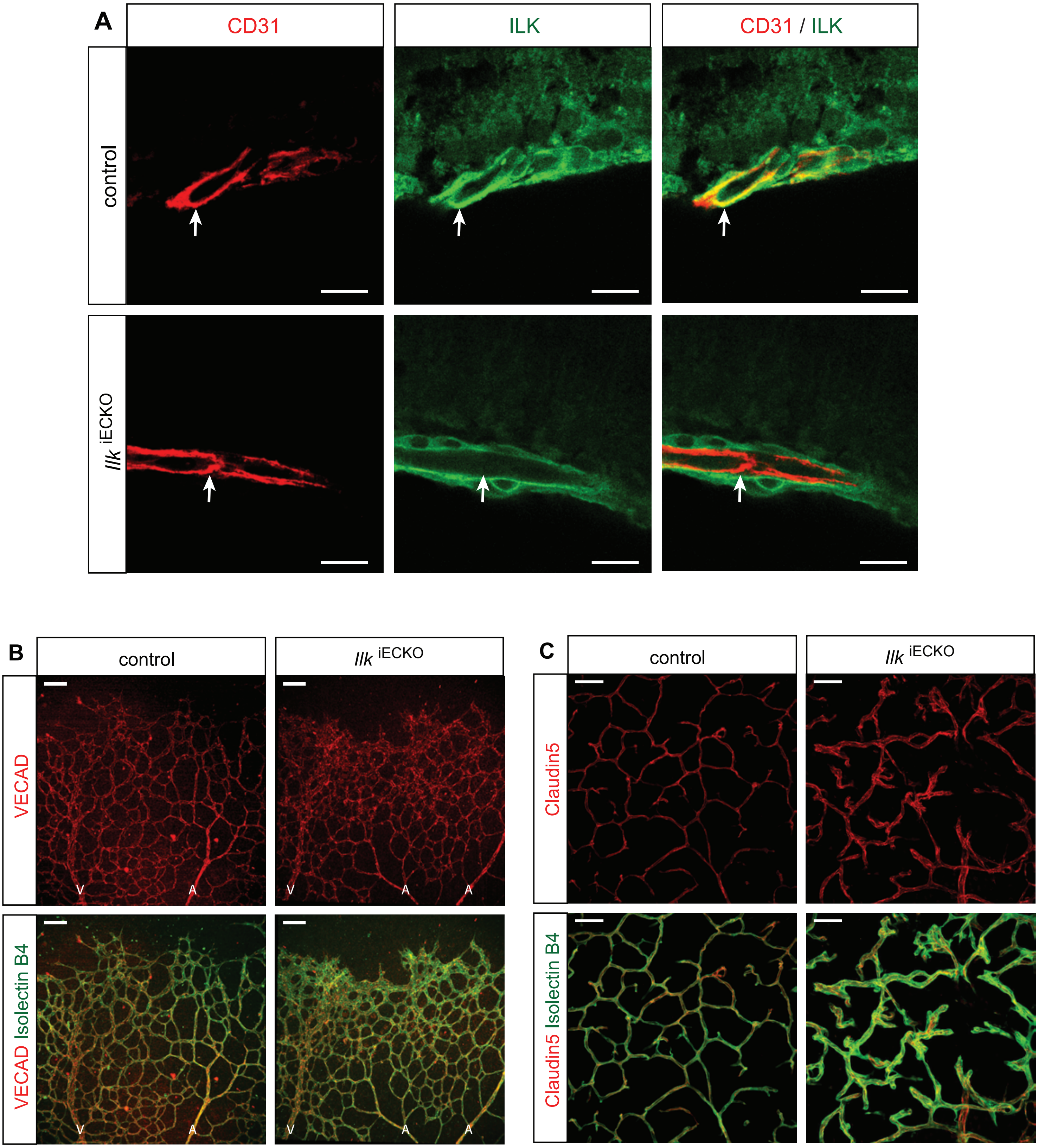

Supplementary Figure 1. EC-specific knockout of *Ilk* and increased junctional complexity in *Ilk* mutant retinal vessels

(A) Confocal image of ILK (green) and CD31 (red) stained sections of P6 retina. ILK expression in CD31 +ECs (white arrows) but not in vessel-associated cells is lost in *Ilk*^{iECKO} mutants. Scale bar, 20 μm.

(B) Overview images of VE-cadherin (red) and isolectin B4 (green) stained P6 control and *Ilk*^{iECKO} retinas. Note dilation of vessels at the mutant angiogenic growth front and increased junctional complexity. Scale bar, 100 μm.

(C) Confocal images of Claudin 5 (red) and isolectin B4 (green) stained P14 control and *Ilk*^{iECKO} retinas. Mutant capillaries are enlarged and tight junction complexity is increased. Scale bar, 50 μm.

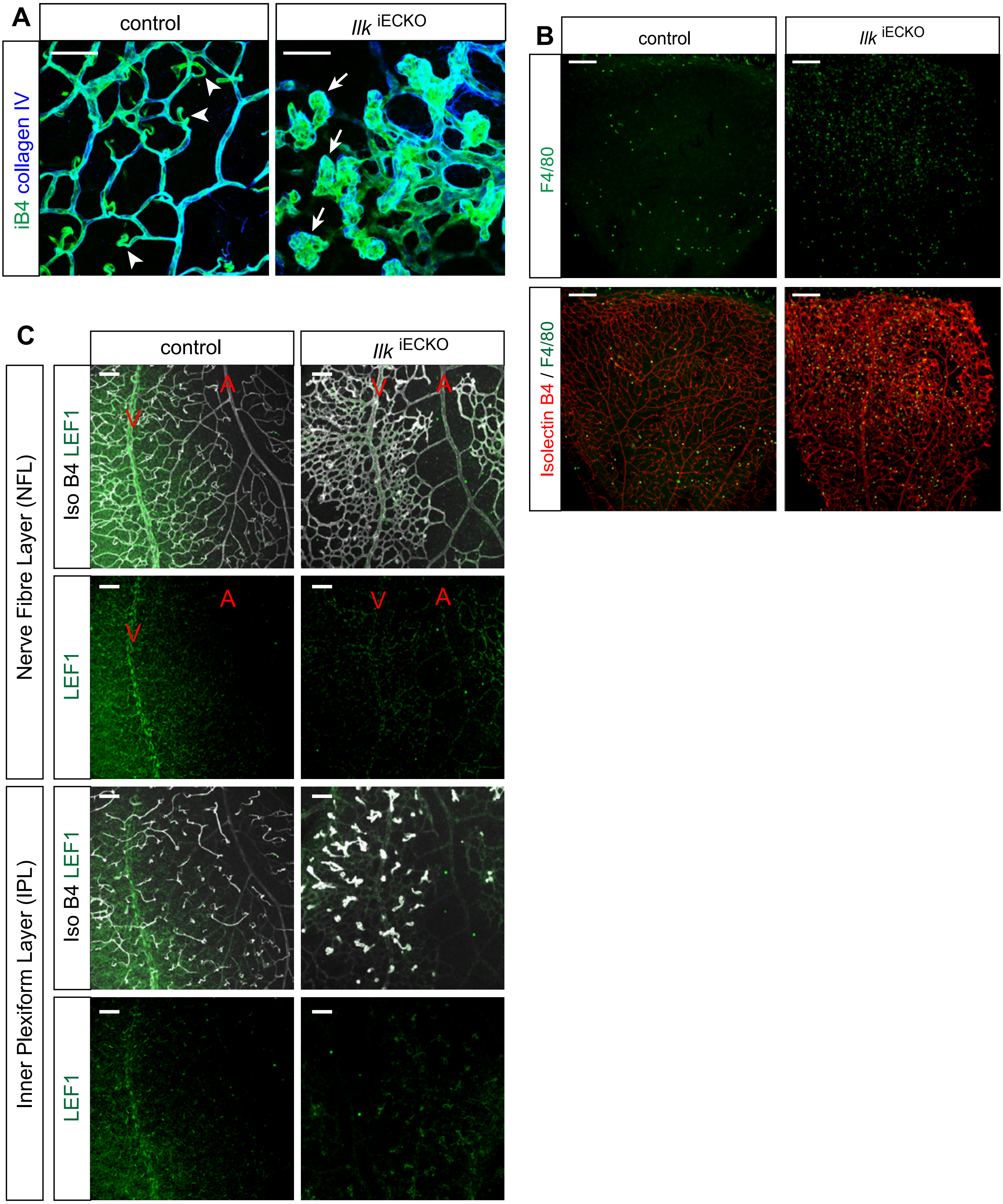

Supplementary Figure 2. Alterations in the *Ilk* mutant retinal vasculature.
(A) Isolectin B4 (green) and collagen type IV (blue) stained retinal vessels at high magnification show vertical branches (arrowheads) in control samples, which are absent and replaced by blind-ended EC clusters (arrows) in *Ilk*^{iECKO} mutants. Scale bar, 50 µm.
(B) F4/80-stained macrophages (green) are increased in P14 *Ilk*^{iECKO} mutants relative to littermate control retinas. Scale bar, 200 µm.
(C) LEF1 (green) immunosignals label venous (V) and capillary ECs but not arteries (A) in the P14 control retina. *Ilk*^{iECKO} mutants show reduced venous LEF1 expression, whereas ectopic signal is seen in arteries. Scale bar, 100 µm.

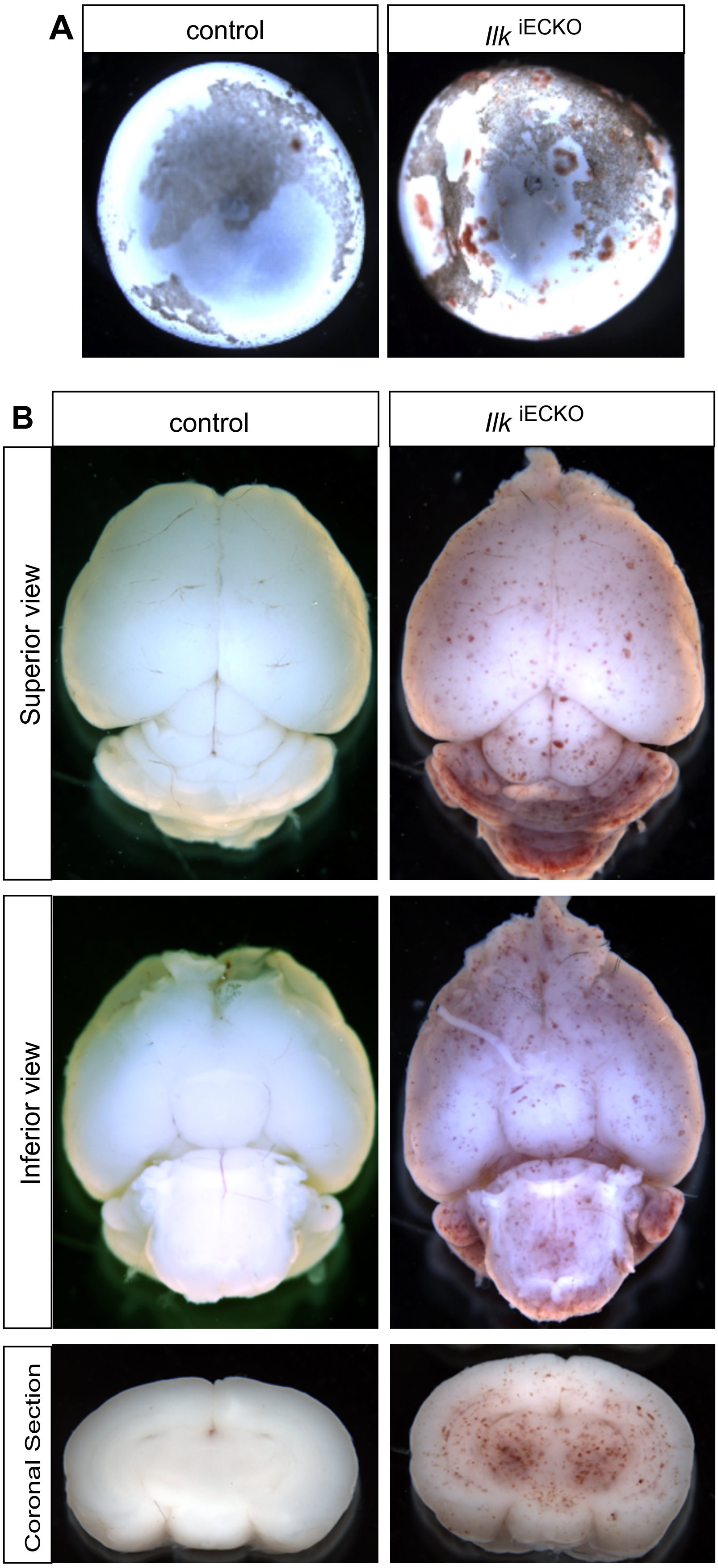

Supplementary Figure 3. Haemorrhaging in the *Ilk* mutant central nervous system.
(A) and **(B)** Haemorrhaging in the P14 *Ilk*^{iECKO} retina **(A)**, cerebral cortex, cerebellum and brain stem of freshly isolated brains **(B)** but not in control samples.

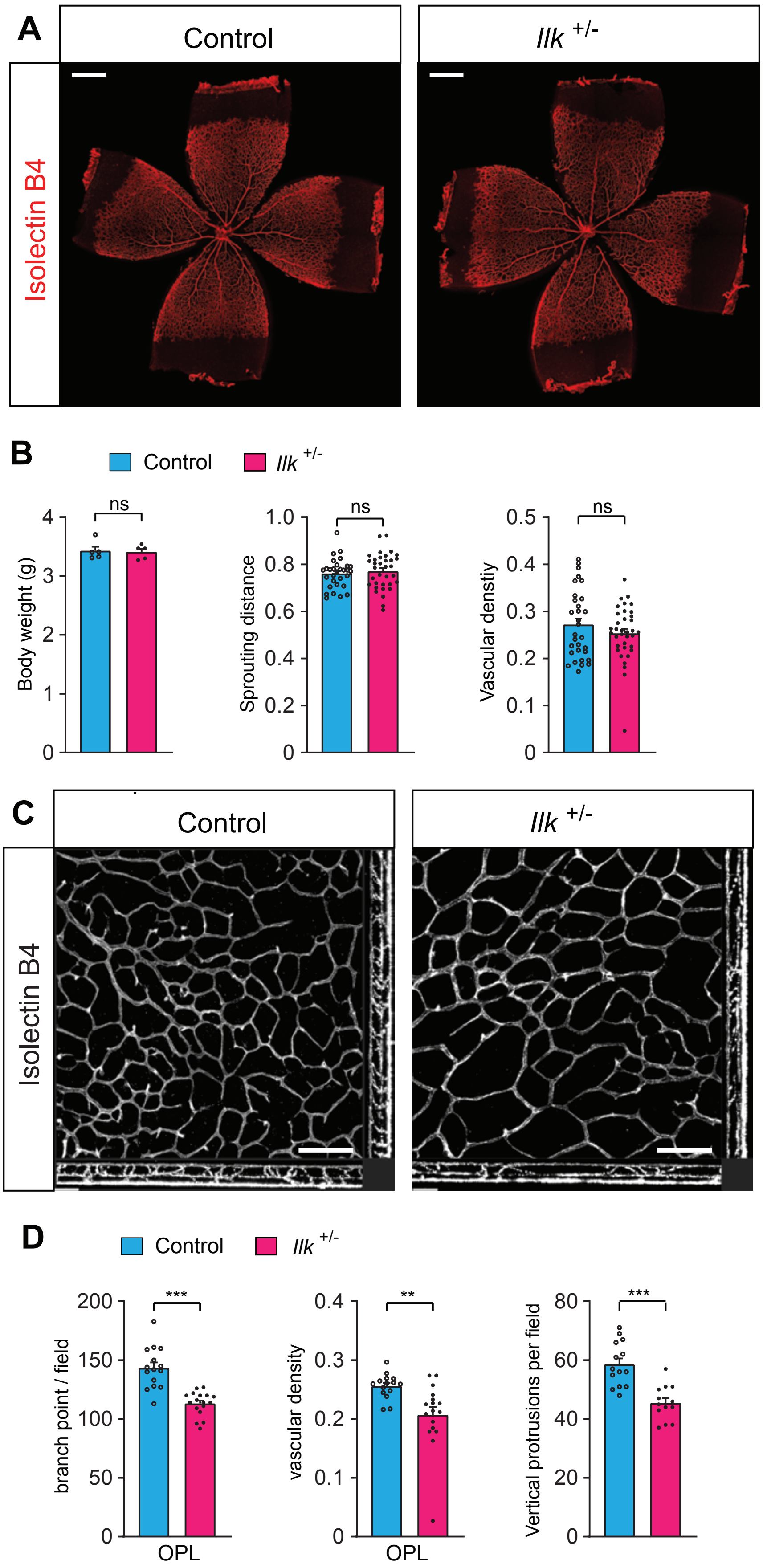

Supplementary Figure 4. Retinal vasculature of *Ilk* heterozygous knockouts.
(A) Isolectin B4-stained whole-mounts of global P6 *Ilk*$^{+/-}$ and control retinas. Scale bar, 500 µm.
(B) Quantification of body weight, vessel density and vascular outgrowth (distance between the central optic nerve and the peripheral edge of retinas). Error bars, s.e.m. p-values (ns: not significant). Student's t-test (n= 7 control and *Ilk*$^{+/-}$ mice for body weight, 8 retinas for each group).
(C) Confocal images of deeper vascular plexus and Z-X/Y projections of isolectin B4 stained control and *Ilk*$^{+/-}$P14 retinas. Scale bar, 100 µm.
(D) Quantification of branch points and vessel density in the deeper vascular plexus and vertical branches per field. Error bars, s.e.m. p-values (***: p<0.001, **: p<0.01), Student's t-test (n=6 retinas). Source data are provided as a Source Data file.

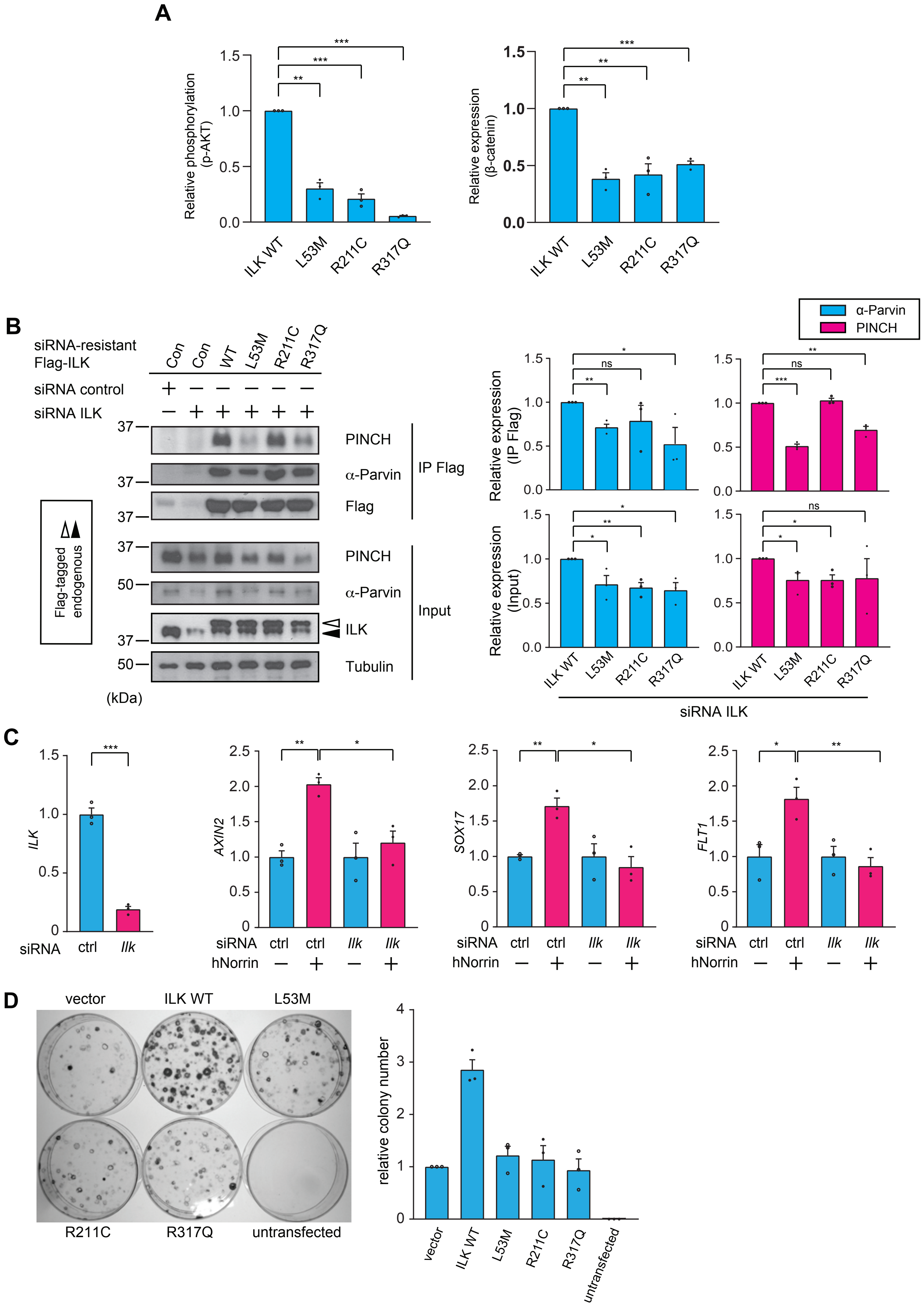

A
Relative phosphorylation (p-AKT)
Relative expression (β-catenin)
1.5
1.0
0.5
0.0
ILK WT
L53M
R211C
R317Q
**
***
B
α-Parvin
PINCH
siRNA-resistant Flag-ILK
Con
Con
WT
L53M
R211C
R317Q
siRNA control
siRNA ILK
Flag-tagged
endogenous
37
50
PINCH
α-Parvin
Flag
IP Flag
ILK
Tubulin
Input
(kDa)
Relative expression (IP Flag)
Relative expression (Input)
ns
*
siRNA ILK
C
ILK
AXIN2
SOX17
FLT1
siRNA
ctrl
Ilk
hNorrin
2.5
2.0
D
vector
ILK WT
L53M
R211C
R317Q
untransfected
relative colony number

Supplementary Figure 5. IPP complex formation with ILK variants.
(A) Quantitation of Western blot data (levels of p-Akt and β-catenin) in Fig. 7A by densitometry. Error bars, s.e.m. p-values (***: $p<0.001$, **: $p<0.01$), Student's t-test (n=3 for each sample).
(B) IP and Western blot analysis of PINCH and α-parvin binding to siRNA-resistant, FLAG-tagged WT or mutant ILK (white arrowhead) after siRNA-mediated knockdown of endogenous *ILK* expression (black arrowhead). Input controls (bottom 4 lanes) show decreased levels of PINCH and α-parvin in the absence of endogenous ILK. PINCH and α-parvin levels are restored by re-expression of FLAG-tagged WT ILK but not by L53M or R317Q ILK. The R211 ILK variant shows better co-immunoprecipitation of PINCH and α-parvin than the two other mutants. Graphs on the right show densitometric quantitation of PINCH and α-parvin bands in total lysates (input) and after IP of tagged ILK. Error bars, s.e.m. p-values (***: $p<0.001$, **: $p<0.01$), Student's t-test (n=3 for each sample).
(C) qPCR analysis of Wnt/β-catenin target gene expression after stimulation with recombinant hNorrin in *ILK* siRNA-treated and control HRMVECs. Error bars, s.e.m. p-values (***: $p<0.001$, **: $p<0.01$, *: $p<0.05$), Student's t-test (n=3).
(D) Colony formation assay with overexpressed WT or mutant ILK to evaluate their transforming activity and quantification of foci number. None of mutants shows comparable colony formation in transfected NIH3T3 cells as ILK WT (n=3).
Source data are provided as a Source Data file.

Figure 7A

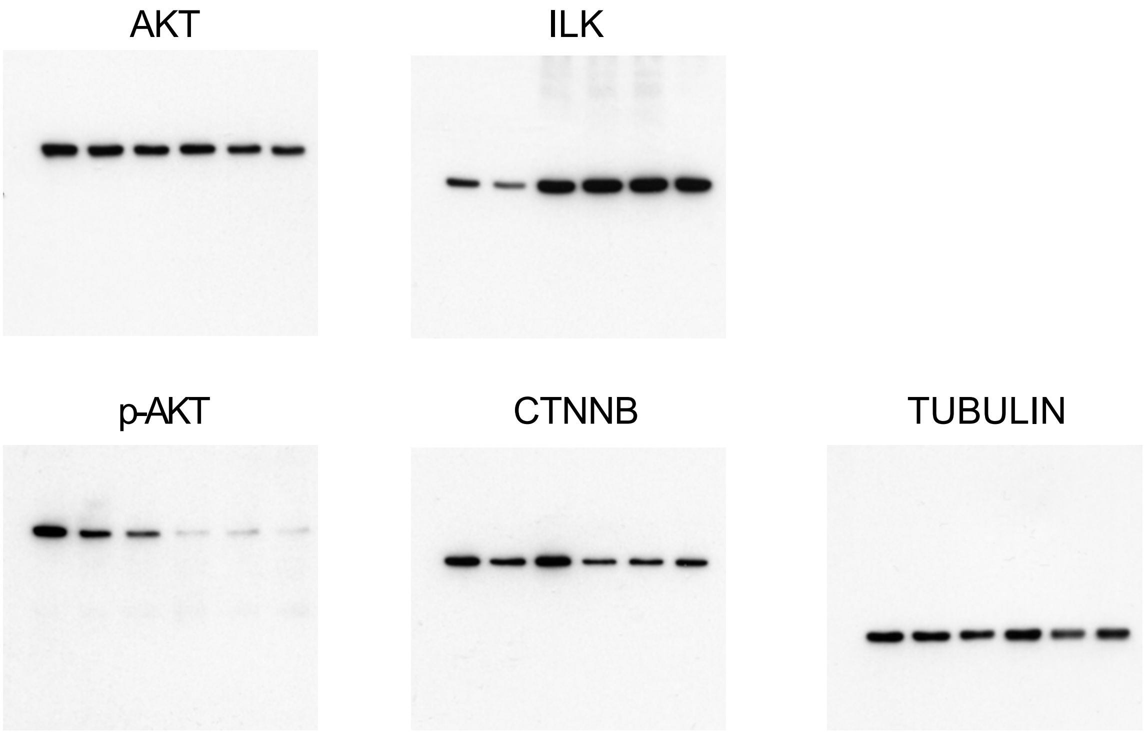

Figure 7C

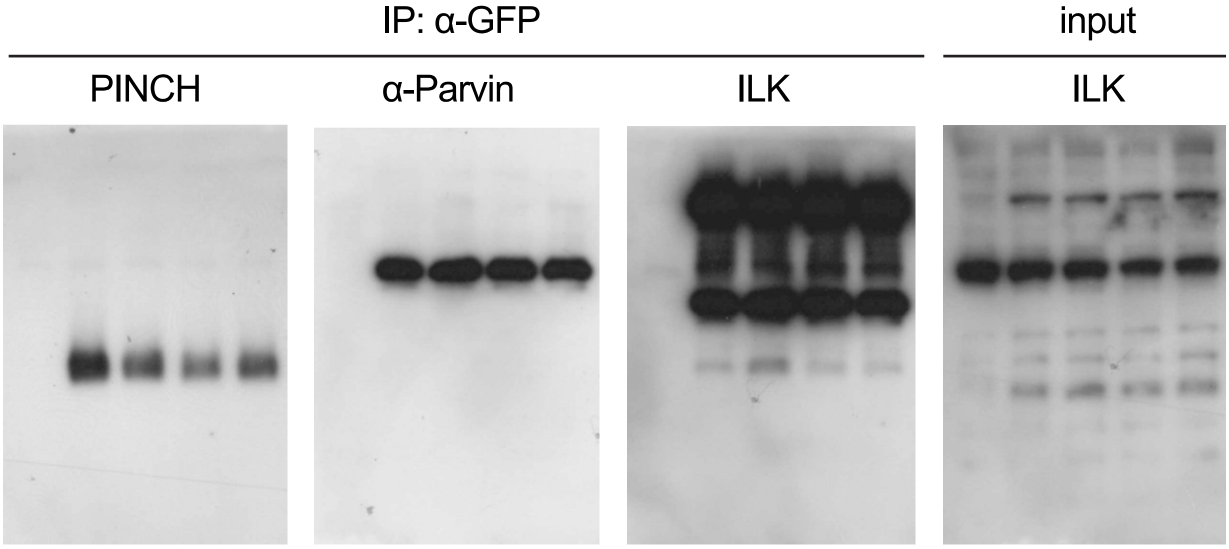

Supplementary Figure 6. Uncropped Western blots related to Figure 7

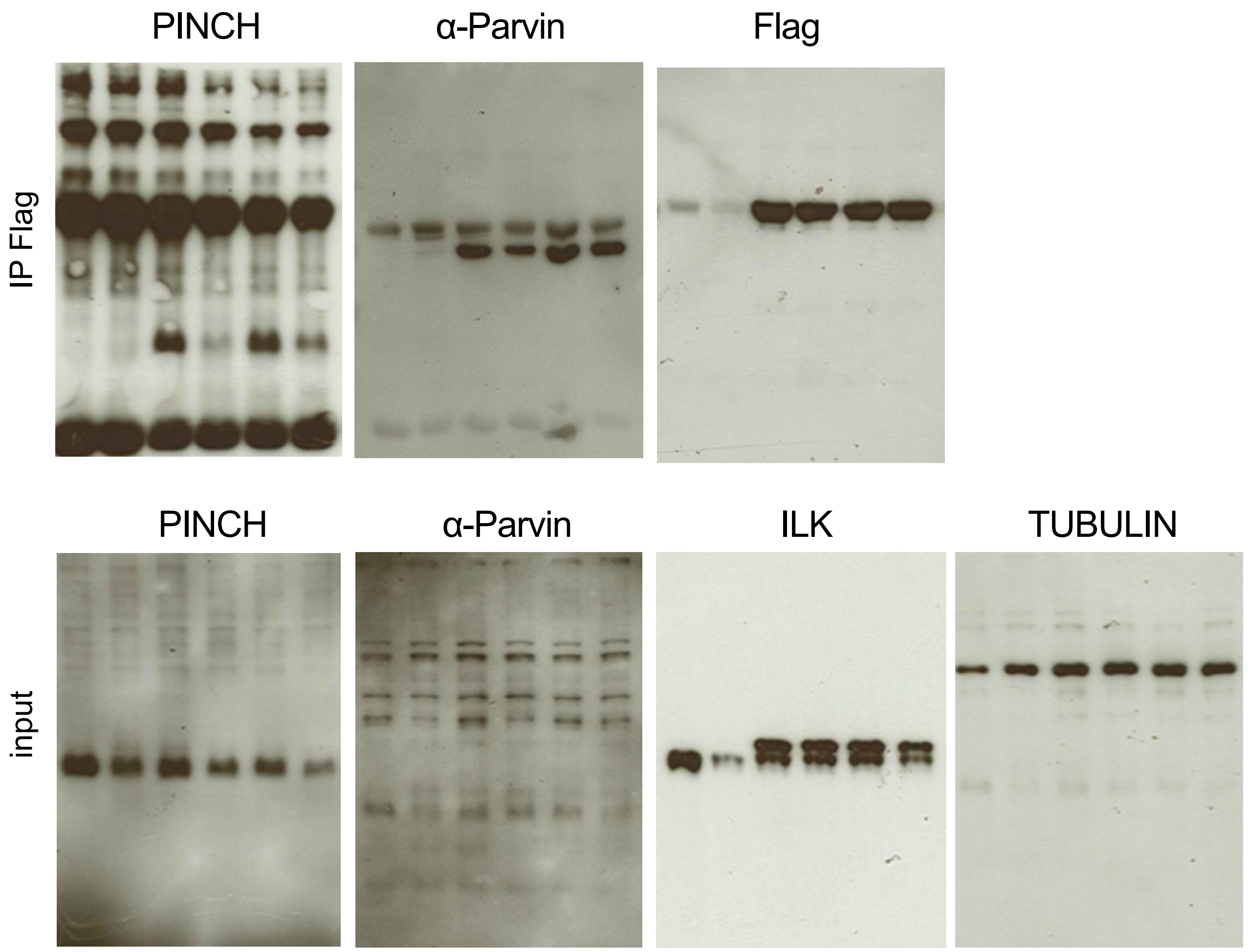

Supplementary Figure 7. Uncropped Western blots related to Supplementary Figure 5

Supplementary Figure 5A

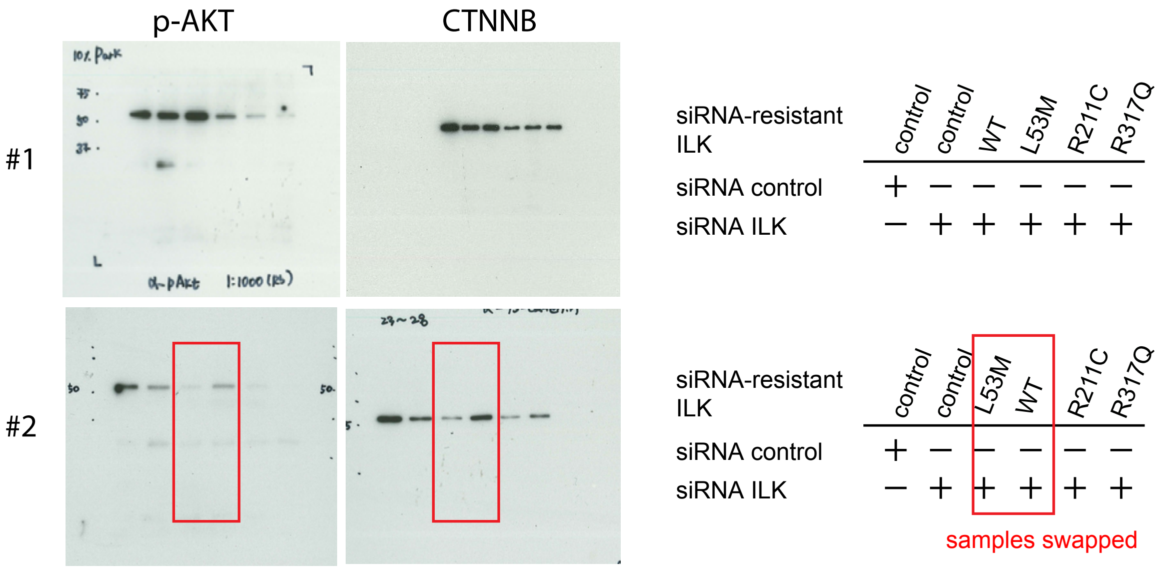

Supplementary Figure 5B

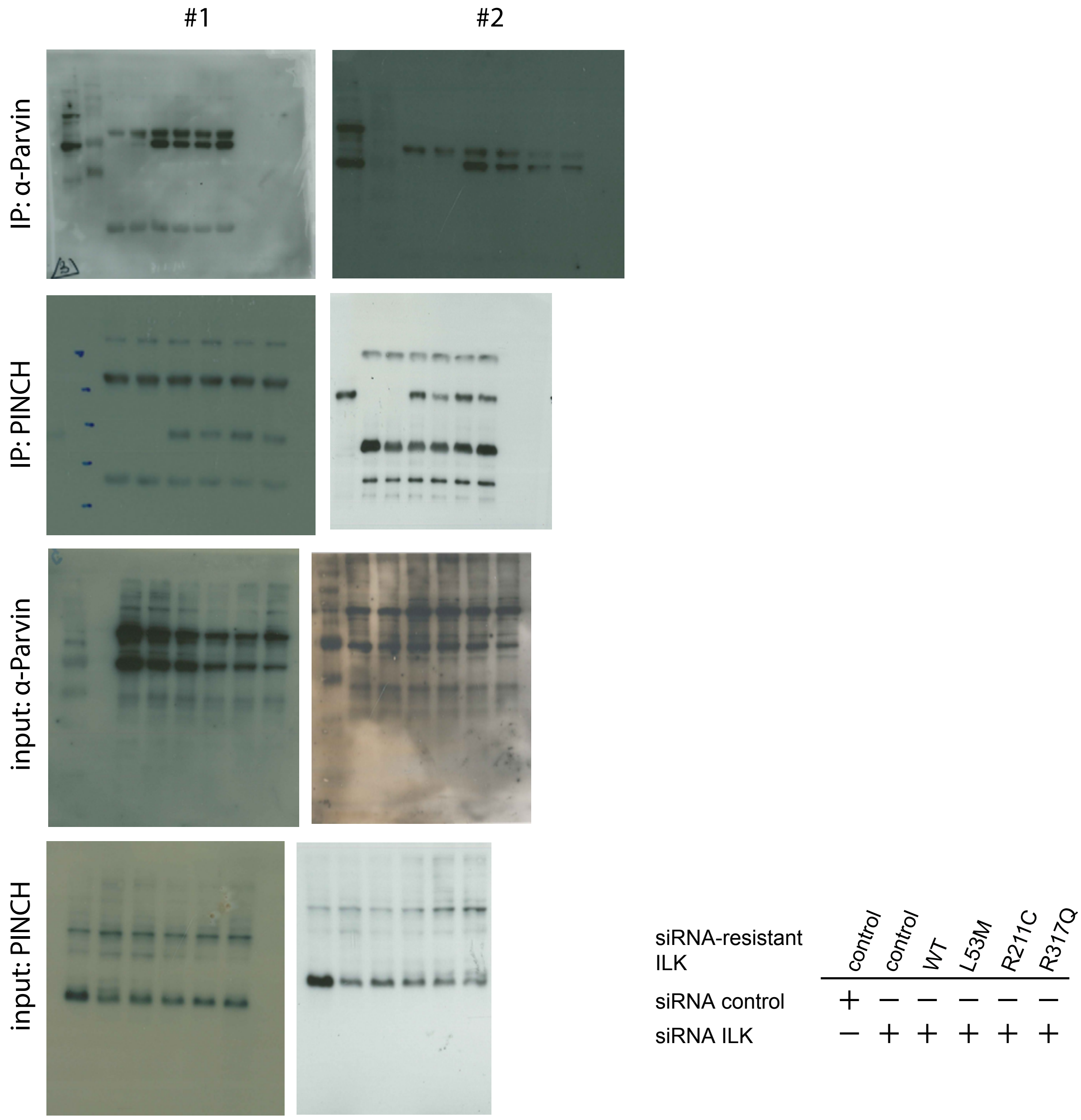

Supplementary Figure 8. Additional uncropped Western blots for quantification shown in Supplementary Figure 5A and 5B
