# Supplementary material for: Integrin-linked kinase controls retinal angiogenesis and is linked to Wnt signaling and exudative vitreoretinopathy
Source: Nat Commun. 2019 Nov 20;10:5243. doi: 10.1038/s41467-019-13220-3 (PMC6868140; doi:10.1038/s41467-019-13220-3)
Supplement: Supplementary file 3 — Reporting Summary [file 41467_2019_13220_MOESM3_ESM.pdf]

nature research

Corresponding author(s): Ralf H. Adams

Last updated by author(s): Jul 11, 2019

Reporting Summary

Nature Research wishes to improve the reproducibility of the work that we publish. This form provides structure for consistency and transparency in reporting. For further information on Nature Research policies, see Authors & Referees and the Editorial Policy Checklist.

Statistics

For all statistical analyses, confirm that the following items are present in the figure legend, table legend, main text, or Methods section.

n/a	Confirmed	
☐	☒	The exact sample size (*n*) for each experimental group/condition, given as a discrete number and unit of measurement
☐	☒	A statement on whether measurements were taken from distinct samples or whether the same sample was measured repeatedly
☐	☒	The statistical test(s) used AND whether they are one- or two-sided<br>*Only common tests should be described solely by name; describe more complex techniques in the Methods section.*
☒	☐	A description of all covariates tested
☐	☒	A description of any assumptions or corrections, such as tests of normality and adjustment for multiple comparisons
☐	☒	A full description of the statistical parameters including central tendency (e.g. means) or other basic estimates (e.g. regression coefficient) AND variation (e.g. standard deviation) or associated estimates of uncertainty (e.g. confidence intervals)
☐	☒	For null hypothesis testing, the test statistic (e.g. *F*, *t*, *r*) with confidence intervals, effect sizes, degrees of freedom and *P* value noted<br>*Give P values as exact values whenever suitable.*
☒	☐	For Bayesian analysis, information on the choice of priors and Markov chain Monte Carlo settings
☒	☐	For hierarchical and complex designs, identification of the appropriate level for tests and full reporting of outcomes
☒	☐	Estimates of effect sizes (e.g. Cohen's *d*, Pearson's *r*), indicating how they were calculated

Our web collection on statistics for biologists contains articles on many of the points above.

Software and code

Policy information about availability of computer code

Data collection	Volocity 6.3, ZEN Software 2.3 SP1 FP1 14.0, Leica Application Suite X 2.0.1.14392, FACS Diva 6.0,
Data analysis	Volocity 6.3, Imaris x64 9.3.0, Photoshop CS6 13.0, Illustrator CS6 16.0.0, Fiji-IJ 1.4.5B, GraphPad Prism 7.0b.

For manuscripts utilizing custom algorithms or software that are central to the research but not yet described in published literature, software must be made available to editors/reviewers. We strongly encourage code deposition in a community repository (e.g. GitHub). See the Nature Research guidelines for submitting code & software for further information.

Data

Policy information about availability of data

All manuscripts must include a data availability statement. This statement should provide the following information, where applicable:

- Accession codes, unique identifiers, or web links for publicly available datasets
- A list of figures that have associated raw data
- A description of any restrictions on data availability

All relevant data supporting the results of the present study are included within the article, its Supplementary Information files and can be obtained from the corresponding author on reasonable request. A Source Data File is included which contains the raw data underlying the reported averages in all Figures and Supplementary Figures.

The following figures that have associated raw data in the Source Data file:

Fig 1E, 1G, 2E, 3A, 4C, 5D, 7E
Supplementary Figure 4B, 4D, 5A-D.

Field-specific reporting

Please select the one below that is the best fit for your research. If you are not sure, read the appropriate sections before making your selection.

☒ Life sciences ☐ Behavioural & social sciences ☐ Ecological, evolutionary & environmental sciences

For a reference copy of the document with all sections, see nature.com/documents/nr-reporting-summary-flat.pdf

Life sciences study design

All studies must disclose on these points even when the disclosure is negative.

Sample size	No statistical methods were used to predetermine sample size; instead sample number was defined according to previous experience and reproducibility of the results across several independent experiments.
Data exclusions	No Samples or animals were excluded from this study.
Replication	Results are based on more than three independent experiments or samples to guarantee reproducibility of the findings. The number of replicates is given in the respective figure legends. And all the raw values are provided at source file.
Randomization	No randomization of mice was used. Randomization is not relevant to our study design. Mice analyzed were littermates.
Blinding	Investigators were not blinded to mouse genotypes during experiments. Data reported for mouse experiments are not subjective but rather based on quantitative analysis. In most instances phenotypic differences are obvious between control and mutant mice.

Reporting for specific materials, systems and methods

We require information from authors about some types of materials, experimental systems and methods used in many studies. Here, indicate whether each material, system or method listed is relevant to your study. If you are not sure if a list item applies to your research, read the appropriate section before selecting a response.

Materials & experimental systems

n/a	Involved in the study	
☐	☒	Antibodies
☐	☒	Eukaryotic cell lines
☒	☐	Palaeontology
☐	☒	Animals and other organisms
☐	☒	Human research participants
☒	☐	Clinical data

Methods

n/a	Involved in the study	
☒	☐	ChIP-seq
☒	☐	Flow cytometry
☒	☐	MRI-based neuroimaging

Antibodies

Antibodies used

All antibodies are commercially purchased and detailed information for them is provided in the Methods section.
The following primary antibodies were used for immunostaining : Isolectin B4 (Vector Labs, B-1205, 1:50), rat anti-VE-cadherin (BD Biosciences, 555289, 1:200), rabbit anti-Collagen IV (Serotec, 2150-1470, 1:200), rat anti-ICAM2 (BD Biosciences, 553326, 1:200), rabbit anti-Claudin5 (Life Technologies, 341600, 1:200), rat anti-Plvap (Developmental Studies Hybridoma Bank, AB 531797, 1:15), rabbit anti ERG (Abcam, ab110639, 1:100) and LEF1 (Cell signaling, #2230, 1:100), ILK (Genetex, GTX62096, 1:100), phospho-AKT (Cell signaling, #4060, 1:100) and β-catenin (Cell signaling, #9562, 1:100) was used for staining.
For the following secondary antibodies (all in 1:400 dilution unless otherwise stated) were used for immunostaining: anti-rabbit IgG conjugated to Alexa Fluor (AF) 488 (Thermo Fisher Scientific, A21206), anti-chicken IgY AF488 (Jackson ImmunoResearch, 703-545-155), anti-rat IgG AF488 (Thermo Fisher Scientific, A21208), anti goat IgG AF488 (Invitrogen, A-11055), anti-rat IgG Cy3 (Jackson ImmunoResearch, 712-165-153), anti-rabbit IgG AF546 (Thermo Fisher Scientific, A10040), anti-goat IgG AF546 (Invitrogen, A-11056), anti-rat IgG AF594 (Thermo Fisher, A21209), anti-rabbit IgG AF594 (Thermo Fisher Scientific, A21207), anti-goat IgG AF594 (Thermo Fisher Scientific, A-11058), anti-rabbit IgG AF647 (Thermo Fisher Scientific, A31573), anti-rat IgG AF647 (Jackson ImmunoResearch, 712-605-153) and anti-goat IgG AF647 (Thermo Fisher Scientific, A21447).
For western : anti-GFP (Abcam, ab6556) or anti-FLAG agarose (MBL, FLA-1), anti-PINCH (BD, 612710, 1: 5,000), anti-?-parvin (Abcam, ab11336, 1: 5,000), anti-ILK (Abcam, ab49979, 1: 5,000), anti-Tubulin (Sigma, T3526, 1: 5,000), anti-FLAG (Sigma, F7425, 1: 5,000), anti-β-catenin (Cell signaling, #8814, 1:1,1000), anti-AKT (Cell signaling, #4691, 1:5,000) and anti-phospho-AKT(ser473) (Cell signaling, #4060, 1:1,000) was used.

Validation

Primary antibodies were validated by the manufacturer and confirmed by specific labeling of target molecules or cell types. Secondary antibodies have been tested in our experimental conditions to rule out unspecific reactivity.

Eukaryotic cell lines

Policy information about cell lines

Cell line source(s)	Human umbrical cord vein endothelial cells are purchased from the ThermoFisher (cat.no. C0035C) and HREC purchased from ACBRI
Authentication	Authentication is proved by company.
Mycoplasma contamination	No Mycoplasma contamination.
Commonly misidentified lines (See ICLAC register)	No misidentified lines were used in this study

Animals and other organisms

Policy information about studies involving animals; ARRIVE guidelines recommended for reporting animal research

Laboratory animals	A full list of the mouse strains used is detailed in the Methods Section. No other laboratory animals were used. Both male and female mice were used in all cases.<br>Pdgfb-icre/ERT2: MGI : 3793852<br>ILK_lox : MGI:2655773<br>Parva_lox: MGI:6272336<br>PGK-Cre: MGI:2178050<br>Age of analysis:<br>PDGFB-iCRE; ILK_lox (homo) : P6 (tamoxifen treatment (tmx) P1-3) / P14 (tmx P3, P5, P7)<br>PDGFB-iCRE; ILK_lox (hetero) : P14 (tmx P3, P5, P7)<br>PDGFB-iCRE; Parva_lox(homo) : P16 (tmx P1-3)<br>PGK-Cre; ILK-lox (hetero) : P6, P14
Wild animals	No wild animals were used in this study.
Field-collected samples	No field-collected samples were used in this study.
Ethics oversight	Animal experiments were performed in compliance with the relevant laws and institutional guidelines, were approved by local animal ethics committees and were conducted with permissions granted by the Landesamt für Natur, Umwelt und Verbraucherschutz (LANUV) of North Rhine-Westphalia or Animal Experiments of the Ludwig-Maximilians University Munich.

Note that full information on the approval of the study protocol must also be provided in the manuscript.

Human research participants

Policy information about studies involving human research participants

Population characteristics	The patients were clinically diagnosed with exudative vitreoretinopathy or a similar disorder. Most of them were sporadic cases and no additional family members were available for segregation analysis of DNA sequence variations.
Recruitment	Patients were selected based on clinical diagnosis.
Ethics oversight	Informed consent according to the declaration of Helsinki was obtained from all subjects (208 patients and 258 controls) involved in genetic testing. The approval for genetic testing was awarded to the Institute of Medical Molecular Genetics by the Federal Office of Public Health (FOPH) in Switzerland. Patients provided informed consent for genetic testing.

Note that full information on the approval of the study protocol must also be provided in the manuscript.
